# A randomized trial of iron isomaltoside 1000 versus oral iron in non-dialysis-dependent chronic kidney disease patients with anaemia

**DOI:** 10.1093/ndt/gfv293

**Published:** 2015-08-06

**Authors:** Philip A. Kalra, Sunil Bhandari, Sanjiv Saxena, Dhananjai Agarwal, Georg Wirtz, Josef Kletzmayr, Lars L. Thomsen, Daniel W. Coyne

**Affiliations:** 1 Salford Royal NHS Foundation Trust, Salford, UK; 2 Hull and East Yorkshire Hospitals NHS Trust, Hull and Hull York Medical School, Kingston Upon Hull, UK; 3 Pushpawati Singhaniya Research Institute, New Delhi, India; 4 SMS Medical College Hospital, Jaipur, Rajasthan, India; 5 Dialysis Centre Kamen, Kamen, Germany; 6 3 Department of Medicine, Socio-Medical Centre East-Danube Hospital, Vienna, Austria; 7 Pharmacosmos A/S, Holbaek, Denmark; 8 Washington University School of Medicine, St. Louis, MO, USA

**Keywords:** chronic kidney disease, iron isomaltoside 1000, iron treatment

## Abstract

**Background:**

Iron deficiency anaemia is common in patients with non-dialysis-dependent chronic kidney disease (NDD-CKD) and is often treated with oral or intravenous (IV) iron therapy. This trial compared the efficacy and safety of IV iron isomaltoside 1000 (Monofer®) and oral iron in NDD-CKD patients with renal-related anaemia.

**Methods:**

The trial was a Phase III open-label, comparative, multicentre, non-inferiority trial conducted in 351 iron-deficient NDD-CKD patients, randomized 2:1 to either iron isomaltoside 1000 (Group A) or iron sulphate administered as 100 mg elemental oral iron twice daily (200 mg daily) for 8 weeks (Group B). The patients in Group A were randomized into A1 (infusion of max. 1000 mg single doses over 15 min) and A2 (bolus injections of 500 mg over 2 min). A modified Ganzoni formula was used to calculate IV iron need. The primary end point was change in haemoglobin concentrations from baseline to Week 4.

**Results:**

Iron isomaltoside 1000 was both non-inferior to oral iron at Week 4 (P < 0.001) and sustained a superior increase in haemoglobin from Week 3 until the end of the study at Week 8 (P = 0.009 at Week 3). The haemoglobin response was more pronounced with iron isomaltoside 1000 doses ≥1000 mg (P < 0.05). Serum-ferritin and transferrin saturation concentrations were also significantly increased with IV iron. Adverse drug reactions were observed in 10.5% in the iron isomaltoside 1000 group and 10.3% in the oral iron group. More patients treated with oral iron sulphate withdrew from the study due to adverse events (4.3 versus 0.9%, P = 0.2).

**Conclusions:**

Iron isomaltoside 1000 was more efficacious than oral iron for increase in haemoglobin and proved to be well tolerated at the tested dose levels in NDD-CKD patients.

## INTRODUCTION

Iron deficiency is a crucial contributor to anaemia in patients with non-dialysis-dependent chronic kidney disease (NDD-CKD). The KDIGO guidelines recommend either treatment with intravenous (IV) iron or alternatively 1–3 months of oral iron therapy in NDD-CKD patients with iron deficiency anaemia based on the severity of iron deficiency, availability of venous access, response to prior oral iron therapy, side effects with prior iron therapy, patient compliance and cost [1]. Oral iron can be inadequately absorbed in CKD patients [1] and is often associated with gastrointestinal adverse effects, and therefore IV iron might improve the patient compliance and treatment success [2].

A previous clinical trial of 626 patients with NDD-CKD and anaemia showed that patients, who were not receiving erythropoiesis-stimulating agents (ESAs), may benefit from IV iron treatment targeting a higher ferritin level. Both IV and oral iron therapy were effective in increasing haemoglobin (Hb), serum ferritin and transferrin saturation (TSAT) levels, but the IV iron (ferric carboxymaltose) therapy group with a higher ferritin target was shown to be superior to oral iron in delaying and/or reducing the requirement for other anaemia management as well as producing a faster haematological response with a greater proportion of patients achieving an Hb increase of ≥1 g/dL [3].

Iron isomaltoside 1000 (Monofer^®^) is a high-dose IV iron licensed for fast infusion. Iron isomaltoside 1000 has previously been shown to be safe and well tolerated and to improve markers of iron deficiency anaemia in patients with CKD [4], chronic heart failure [5] and inflammatory bowel disease [6].

The aim of this comparative trial was to evaluate the efficacy and short-term safety of iron isomaltoside 1000 administered as a single bolus or split bolus injection compared with oral iron sulphate in patients with NDD-CKD and renal-related anaemia. The primary objective was to compare IV iron isomaltoside 1000 with oral iron sulphate in reducing renal-related anaemia in patients with NDD-CKD, evaluated as the ability to increase Hb.

## MATERIALS AND METHODS

### Trial design

This prospective, randomized, comparative, open-label, non-inferiority, multicentre trial was conducted from June 2010 to April 2014. The patients attended seven visits: screening visit (Visit 1), baseline (Visit 2), four on-treatment and follow-up visits (Visit 3–6) and one end-of-trial visit (Visit 7) during an 8-week period.

The protocol and amendments were approved by local ethics committees/Institutional Review Boards and competent authorities (EudraCT number: 2009-016728-29). The trial was conducted in accordance with good clinical practice and the Declaration of Helsinki. The trial was registered at ClinicalTrials.gov (NCT01102413) on 26 March 2010. Written informed consent was obtained prior to any trial-related activities.

### Participants

The trial took place at 67 sites (hospitals or private dialysis clinics) on 3 continents: 17 in India, 10 in Germany, 7 in UK, 7 in Austria, 7 in Russia, 5 in Poland, 4 in Denmark, 3 in Romania, 3 in USA, 2 in Sweden and 2 in Ireland. Patients who were ≥18 years of age with estimated glomerular filtration rate (eGFR) between 15 and 59 mL/min/1.73 m^2^, Hb <11.0 g/dL, either or both serum ferritin <200 μg/L and TSAT <20% and had not received ESA treatment within 8 weeks prior to screening were eligible to participate. The full inclusion and exclusion criteria are shown in Table 1.

**Table 1.** Inclusion and exclusion criteria

Inclusion criteria
Patients ≥18 years of age with NDD-CKD with MDRD calculated eGFR between 15 and 59 mL/min/1.73 m^2^ Hb <11.0 g/dL (6.80 mmol/L) Either or both of the following iron stores indicators below target (s-ferritin <200 μg/L and TSAT <20%) Life expectancy beyond 12 months by principal investigator's (PI's) judgement Willingness to participate after signing informed consent and any authorization as required by local law (e.g. protected health information for North America)
Exclusion criteria
Anaemia predominantly caused by factors other than renal impairment or iron deficiency (according to PI's judgment) Iron overload or disturbances in utilization of iron (e.g. haemochromatosis and haemosiderosis) Drug hypersensitivity (i.e. previous hypersensitivity to iron dextran or iron mono- or disaccharide complexes or iron sulphate or any excipients of the study drug) History of multiple allergies Decompensated liver cirrhosis or active hepatitis (alanine aminotransferase more than three times upper normal limit) Active acute or chronic infections (assessed by clinical judgement), supplied with white blood cells and C-reactive protein Rheumatoid arthritis with symptoms or signs of active joint inflammation Pregnancy or nursing. In order to avoid pregnancy, women had to be post-menopausal (at least 12 months since last menstruation), surgically sterile or women of child bearing potential must have used one of the following contraceptives during the whole study period and after the study had ended for at least five times plasma biological half-life of the investigational medicinal product (5 days): contraceptive pills, intrauterine devices, contraceptive depot injections (prolonged-release gestagen), subdermal implantation, vaginal ring and transdermal patches Extensive active bleeding necessitating blood transfusion Planned elective surgery during the study Participation in any other clinical study within 3 months prior to screening Known intolerance to oral iron treatment Untreated vitamin B_12_ or folate deficiency IV or oral iron treatment or blood transfusion within 4 weeks prior to screening visit ESA treatment within 8 weeks prior to screening visit s-Ferritin >500 µg/L Any other medical condition that, in the opinion of the PI, may have caused the patient to be unsuitable for the completion of the study or placed the patient at potential risk from being in the study or interfere with study drug evaluation (e.g. uncontrolled hypertension, unstable ischaemic heart disease or uncontrolled diabetes mellitus) Body weight <30 kg

### Interventions

Patients were randomized 2:1 to either iron isomaltoside 1000 (Group A) or oral iron sulphate (Group B). The total IV iron needed for each patient in Group A was calculated according to an adapted Ganzoni formula: cumulative iron dose (mg) = [body weight (kg) × (13 g/dL − actual Hb (g/dL))] × 2.4 + depot iron (set at 500 mg) [7]. Patients treated with iron isomaltoside 1000 either received an IV infusion (Group A1) of maximum 1000 mg iron isomaltoside 1000 as single doses over 15 min (full iron replacement was achieved by one or up to two doses at a weekly interval) or IV bolus injections (Group A2) of 500 mg iron isomaltoside 1000 administered over 2 min once weekly until full replacement dose was achieved. Patients who received oral iron sulphate (Group B) were treated daily for 8 weeks with 200 mg given as 100 mg twice a day.

During the trial, the patients were prohibited from having any other iron supplementation, blood transfusion, ESAs and medications that would potentially yield a decrease in oral iron absorption.

### Objective and outcomes

The trial was designed with the primary objective to demonstrate non-inferiority of iron isomaltoside 1000 when compared with oral iron. The primary efficacy outcome was change in Hb concentrations from baseline to Week 4. The secondary efficacy outcome included change in Hb concentration from baseline to Weeks 2 and 8; change in concentrations of serum-iron, serum-ferritin, TSAT and total iron-binding capacity (TIBC) from baseline to Weeks 1, 2, 4 and 8 and change in total quality-of-life (QoL) score (Linear Analogue Scale Assessment) from baseline to Weeks 4 and 8. The LASA questionnaire is a widely used brief measurement tool, consisting of three questions that evaluate energy level, daily activity and overall QoL [8].

The safety outcomes of the trial were to determine the number of patients who experienced any adverse drug reaction (ADR), including any serious adverse reaction (SAR), and safety laboratory assessments. The nature and causality of the adverse events and ADRs were objectively assessed by a trial safety committee. The primary outcome was tested for non-inferiority, whereas the remaining outcomes were tested for superiority.

### Sample size and randomization

A stratified block randomization methodology was used in the trial to assign patients in a 1:1:1 ratio (2:1 randomization to Groups A and B) to receive either iron isomaltoside 1000 as 1000 mg infusions (Group A1), or iron isomaltoside 1000 as 500 mg bolus injections (Group A2), or oral iron sulphate (Group B). The block size was 6. An interactive web response system (IWRS) was used to randomize the patients. When the patient data had been entered into the IWRS, a unique randomization number was generated which identified the treatment the patient was allocated to. The randomization was stratified by whether the patients received IV iron treatment in the past or not and whether the current Modification of Diet in Renal Disease (MDRD) calculated eGFR (women: 175 × (creatinine concentration exp[−1.154]) × (age exp[−0.203]) × 0.742; men: 175 × (creatinine concentration exp[−1.154]) × (age exp[−0.203])) [9] was between 15 and 45 mL/min/1.73 m^2^ or 46 and 59 mL/min/1.73 m^2^.

The screening and enrolment of the patients were performed by the investigator at the site, whereas the entering of the patient data into the IWRS generating the randomization number was typically performed by the trial nurse or trial coordinator.

Patients and investigators were not blinded to trial medications during the study; however, since the primary outcome was a laboratory measurement, it was felt that this would not be affected by the open-label trial design.

With a 2:1 randomization, a two-sided significance level of 5% and a non-inferiority margin of −0.5 g/dL, there was 80% power to demonstrate non-inferiority with 214 patients in Group A and 107 patients in Group B. As the trial was designed to demonstrate non-inferiority, and it was a requirement that the analyses of the full analysis set (FAS) and the per protocol (PP) population should lead to similar conclusions, both analysis sets needed to be powered properly. It was anticipated that ∼10% of patients would have major protocol violations, and so a total of 350 patients were to be randomized.

No interim analysis of efficacy parameters was performed, but s-phosphate was analysed when 25, 50 and 100 patients had been exposed to iron isomaltoside 1000. The analysis of s-phosphate was not related to the non-inferiority hypothesis, but only part of monitoring safety in the trial.

### Statistical methods

**FIGURE 1:**
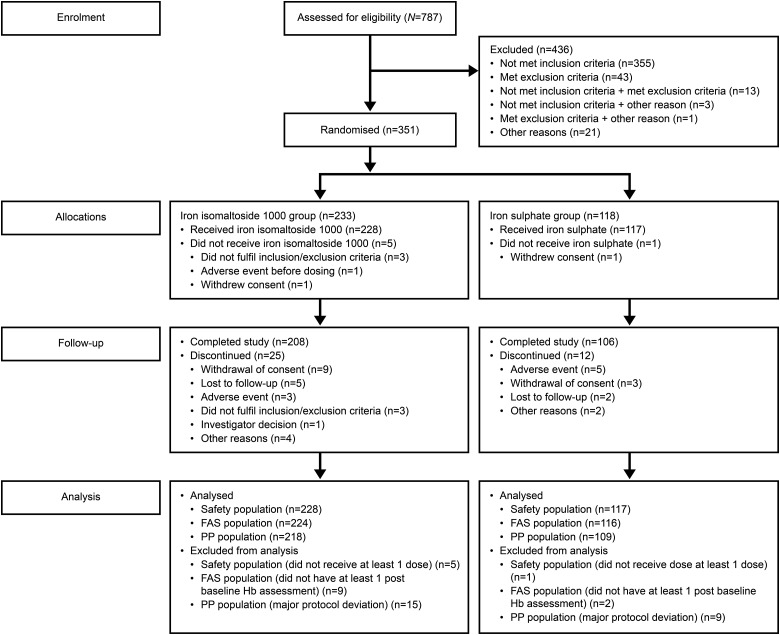
Patient disposition.

The following data sets were used in the analyses (Figure 1).

The randomized population (*N* = 351) included all patients who were randomized in the trial. The safety population (*N* = 345) included all patients who were randomized and received at least one dose of the trial drug. The FAS population (*N* = 340) included all patients who were randomized into the trial, received at least one dose of the trial drug and had at least one post-baseline Hb assessment. The PP population (*N* = 327) included all patients in the FAS who did not have any major protocol deviation of clinical or statistical relevance.

The primary efficacy analyses were conducted on FAS and PP populations, secondary efficacy analyses on the FAS population and the safety analysis was conducted on the safety population.

The primary efficacy data were tabulated using number, mean, SD, minimum, maximum and 95% confidence interval (CI). A mixed model for repeated measures (MMRM) was used to compare the average change in Hb concentration from baseline to end-of-study visit with the inclusion of treatment, visit, treatment × visit interactions, country and stratum [past treatment with parenteral iron (yes/no) and current eGFR between 15 and 45 mL/min/1.73 m^2^ or 46 and 59 mL/min/1.73 m^2^] as factors and baseline Hb as covariate. The treatment difference at Week 4 was derived from the interaction between treatment and visit.

The primary analysis was to assess non-inferiority and the non-inferiority margin was set as −0.5 g/dL. This margin was in line with previous studies and was regarded as clinically relevant. If the 95% CI lay entirely above 0, this was considered evidence of superiority in terms of statistical significance at the 5% level. In that case, the P-value associated with a test of superiority was calculated and an evaluation of whether this was sufficiently small to reject the hypothesis of no difference was undertaken. The secondary objectives were to assess other relevant haematology parameters, the effect on QoL and safety.

The MMRM was used to compare the average change in Hb concentration from baseline to Weeks 2 and 8 with inclusion of treatment, visit, treatment × visit interactions, country and stratum [past treatment with parenteral iron (yes/no) and current eGFR between 15 and 45 mL/min/1.73 m^2^ or 46 and 59 mL/min/1.73 m^2^] as factors and baseline Hb as covariate. The treatment difference at the relevant weeks was derived from the interaction between treatment and visit. The same method was followed to compare the between-treatment group change in serum-iron, serum-ferritin, TSAT and TIBC from baseline to Week 1, 2, 4 or 8 and in QoL score from baseline to Week 4 or 8.

The baseline characteristics and safety data were displayed descriptively. All tests were two tailed and the significance level was 0.05.

## RESULTS

### Patients

A total of 743 patients were screened in the period 30 June 2010 to 24 February 2014 of whom 351 patients were randomized 2:1 into Group A (233 patients) and Group B (118 patients). Group A was further divided into subgroups A1 (infusion dose, 117 patients) and A2 (split dose, 116 patients). The last patient's last visit was 25 April 2014.

Out of the 351 patients enrolled, 314 (89.5%) patients completed the trial and 37 (10.5%) patients discontinued. The details of patient disposition are summarized in Figure 1.

The patient demographics and baseline characteristics are summarized in Table 2 and baseline laboratory parameters are shown in Table 3. Overall baseline characteristics in Groups A and B were comparable between the treatment groups (Tables 2 and 3). For patients in Group A, the gender distribution was 39.5% men and 60.5% women, and mean (SD) age was 58 (16) years, Hb 9.67 (1.13) g/dL, serum ferritin 94.99 (112.79) µg/L, TSAT 18.10 (27.45) % and eGFR 27.06 (10.66) mL/min/1.73 m^2^. In Group B, the data were 54.2% men and 45.8% women, age 58 (16) years, Hb 9.64 (1.05) g/dL, serum ferritin 98.81 (90.19) µg/L, TSAT 15.51 (7.76) % and eGFR 27.05 (10.50) mL/min/1.73 m^2^.

**Table 2.** Summary of baseline demographics for treatment allocation (randomized population)

Statistics/category	Treatment group		
	Iron isomaltoside 1000 (*n* = 233)	Iron sulphate (*n* = 118)	Overall (*N* = 351)
Age (years)			
*n*	232	118	350
Mean (SD)	57.63 (15.54)	57.94 (16.34)	57.73 (15.79)
Median (min.:max.)	58.00 (22:93)	57.50 (20:90)	58.00 (20:93)
Gender, *n* (%)			
Men	92 (39.5)	64 (54.2)	156 (44.4)
Women	141 (60.5)	54 (45.8)	195 (55.6)
Ethnic origin, *n* (%)			
Caucasian	87 (37.3)	47 (39.8)	134 (38.2)
Black	–	1 (0.8)	1 (0.3)
Asian	139 (59.7)	64 (54.2)	203 (57.8)
Others	6 (2.6)	6 (5.1)	12 (3.4)
BMI (kg/m^2^)			
*n*	232	118	350
Mean (SD)	25.80 (6.66)	25.27 (6.60)	25.62 (6.64)
Median (min.:max.)	24.86 (13.33:52.23)	24.55 (13.92:51.68)	24.81 (13.33:52.23)

**Table 3.** Baseline laboratory parameters, FAS

Statistics/category	Treatment group			
	Iron isomaltoside 1000 infusion (*n* = 114)	Iron isomaltoside 1000 bolus (*n* = 110)	All iron isomaltoside 1000 (*n* = 224)	Iron sulphate (*n* = 116)
Hb (g/dL)				
*n*	114	110	224	116
Mean (SD)	9.73 (1.09)	9.60 (1.17)	9.67 (1.13)	9.64 (1.05)
Median (min.:max.)	9.90 (6.5:12.1)	9.80 (5.2:11.7)	9.80 (5.2:12.1)	9.80 (6.7:11.5)
Serum iron (µmol/L)				
*n*	114	110	224	116
Mean (SD)	11.23 (18.82)	9.63 (6.86)	10.44 (14.26)	8.93 (4.01)
Median (min.:max.)	8.86 (0.5:196.0)	8.77 (1.8:65.5)	8.77 (0.5:196.0)	8.59 (1.8:23.8)
Serum ferritin (µg/L)				
*n*	114	110	224	116
Mean (SD)	80.18 (114.33)	110.35 (109.58)	94.99 (112.79)	98.81 (90.19)
Median (min.:max.)	47.30 (3.0:955.4)	79.05 (3.6:609.3)	60.85 (3.0:955.4)	78.95 (3.1:550.0)
TSAT (%)				
*n*	114	110	224	116
Mean (SD)	19.20 (36.79)	16.97 (11.67)	18.10 (27.45)	15.51 (7.76)
Median (min.:max.)	13.56 (0.7:388.0)	15.56 (3.0:99.5)	14.38 (0.7:388.0)	14.00 (2.5:39.7)
TIBC (µmol/L)				
*n*	114	110	224	116
Mean (SD)	61.71 (13.62)	57.08 (13.42)	59.44 (13.69)	57.70 (13.93)
Median (min.:max.)	59.52 (38.1:105.8)	54.06 (34.7:111.2)	56.74 (34.7:111.2)	56.47 (28.5:108.5)
eGFR (mL/min/1.73 m^2^)				
*n*	114	110	224	116
Mean (SD)	26.77 (10.64)	27.35 (10.72)	27.06 (10.66)	27.05 (10.50)
Median (min.:max.)	23.00 (15:57)	24.00 (15:56)	24.00 (15:57)	24.00 (15:58)
C-reactive protein (mg/L)				
*n*	114	110	224	116
Mean (SD)	8.64 (30.76)	9.38 (15.83)	9.00 (24.54)	8.55 (13.31)
Median (min.:max.)	3.00 (0.20:316.68)	3.60 (0.20:100.74)	3.16 (0.20:316.68)	3.36 (0.22:80.0)

Conversion factor for serum iron: µmol/L/0.179 = µg/dL.

### Exposure to iron

In Group A1, 116 patients were dosed with iron isomaltoside 1000 at baseline (mean ± SD: 884 ± 125 mg, range: 750–1000 mg) and 9 were dosed again at Week 1 (306 ± 110 mg, range: 250–500 mg). In Group A2, 112 patients were dosed with iron isomaltoside 1000 at baseline (506 ± 53 mg, range: 440–1000 mg), 107 patients were dosed again at Week 1 (393 ± 138 mg, range: 250–1000 mg) and 16 patients were dosed again at Week 2 (313 ± 112 mg, range: 250–500 mg). In Group B, 117 patients were dosed with 200 mg iron sulphate daily for 8 weeks (11 200 mg in total). There were minor protocol deviations regarding the dosing regimen. If the administered dose was not exactly the calculated dose but was within the range 80–120%, this was regarded as a minor deviation, whereas if the administered dose was outside this range, it was considered as a major deviation and the patient was excluded from the PP analysis.

### Efficacy results

#### Change in Hb concentration

The primary analysis (change in Hb from baseline to Week 4) was conducted on the FAS (*N* = 340) and PP analysis set (*N* = 327).

**FIGURE 2:**
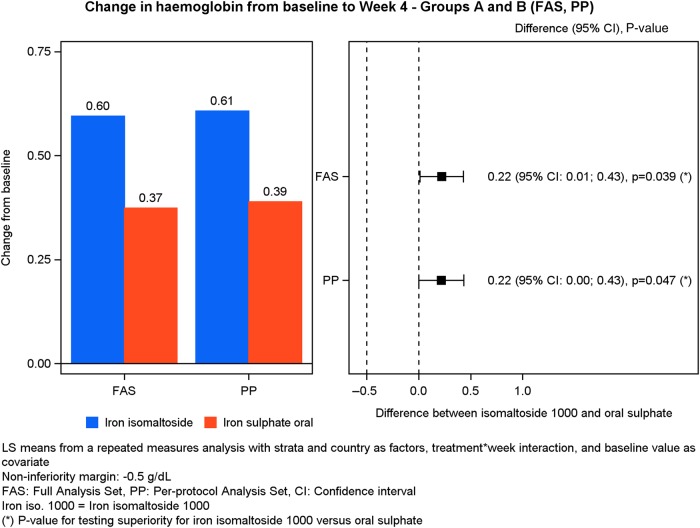
Change in Hb from baseline to Week 4.

The test for non-inferiority showed that iron isomaltoside 1000 was non-inferior to iron sulphate in its ability to increase Hb from baseline to Week 4 in both the FAS and PP data sets (FAS—difference estimate: 0.22, 95% CI: 0.012; 0.43, P < 0.001; PP—difference estimate: 0.22, 95% CI: 0.003; 0.43, P < 0.001) (Table 4, Figure 2). Similar results were observed when the iron isomaltoside 1000 infusion and bolus subgroups (Groups A1 and A2) were compared with the iron sulphate group (Group B) in both FAS and PP data sets. For the FAS, the difference estimate for A1 versus B was 0.27, 95% CI: 0.015; 0.53, P < 0.001, and for A2 versus B, it was 0.17, 95% CI: −0.055; 0.39, P < 0.001. For the PP data set, the difference estimate for A1 versus B was 0.27, 95% CI: 0.007; 0.54, P < 0.001, and for A2 versus B, it was 0.16, 95% CI: −0.066; 0.39, P < 0.001 (Supplementary Tables S1 and S2). In addition, iron isomaltoside 1000 showed superiority over iron sulphate in terms of a significantly higher increase in Hb concentration from baseline to Week 4 (FAS: P = 0.039; PP: P = 0.047). A similar result was observed when Group A1 was compared with Group B, whereas there was no superiority between Groups A2 and B (Supplementary Tables S1 and S2).

**Table 4.** Laboratory parameters: estimated effect size and its precision, Group A versus B

Laboratory parameter, time point (number of patients)	Iron isomaltoside 1000 (Group A), least-square mean estimate^a^	Iron sulphate (Group B), least-square mean estimate^a^	Difference estimates (95% CI)	P value
Hb (g/dL)—FAS				
Week 2 (Group A: 210, Group B: 110)	0.33	0.27	0.059 (−0.11; 0.23)	0.49
Week 4 (Group A: 209, Group B: 108)	0.60	0.37	0.22 (0.012; 0.43)	<0.001/0.039^b^
Week 8 (Group A: 210, Group B: 112)	0.94	0.49	0.45 (0.20; 0.69)	<0.001
Hb (g/dL)—PP analysis set				
Week 4 (Group A: 204, Group B: 106)	0.61	0.39	0.22 (0.003; 0.43)	<0.001/0.047^b^
Serum iron (µg/dL)—FAS				
Week 1 (Group A: 217, Group B: 109)	6.22	2.66	3.55 (1.80; 5.31)	<0.001
Week 2 (Group A: 209, Group B: 110)	4.05	2.02	2.03 (0.72; 3.34)	0.003
Week 4 (Group A: 208, Group B: 108)	2.89	1.99	0.90 (−0.31; 2.10)	0.14
Week 8 (Group A: 209, Group B: 112)	2.81	1.86	0.95 (−0.16; 2.06)	0.091
Serum ferritin (ng/mL)—FAS				
Week 1 (Group A: 217, Group B: 109)	353	32	321 (270; 373)	<0.001
Week 2 (Group A: 209, Group B: 110)	387	52	335 (252; 418)	<0.001
Week 4 (Group A: 208, Group B: 108)	289	54	235 (170; 301)	<0.001
Week 8 (Group A: 209, Group B: 112)	222	66	156 (105; 206)	<0.001
TSAT (%)—FAS				
Week 1 (Group A: 217, Group B: 109)	11.01	2.64	8.37 (5.17; 11.57)	<0.001
Week 2 (Group A: 209, Group B: 110)	8.34	2.14	6.20 (3.86; 8.54)	<0.001
Week 4 (Group A: 208, Group B: 108)	7.34	2.57	4.77 (2.45; 7.08)	<0.001
Week 8 (Group A: 209, Group B: 112)	6.73	3.54	3.20 (1.06; 5.33)	0.004
TIBC (µmol/L)—FAS				
Week 1 (Group A: 217, Group B: 109)	−4.64	−0.72	−3.92 (−5.43; −2.41)	<0.001
Week 2 (Group A: 209, Group B: 110)	−7.52	−2.33	−5.18 (−7.01; −3.36)	<0.001
Week 4 (Group A: 208, Group B: 108)	−9.85	−2.70	−7.16 (−8.99; −5.32)	<0.001
Week 8 (Group A: 209, Group B: 112)	−9.97	−5.19	−4.78 (−6.35; −3.20)	<0.001

Conversion factor for serum iron: µmol/L/0.179 = µg/dL.

^a^Least-square means from repeated measures model with treatment, visit, treatment × visit interactions, country and stratum [past treatment with parenteral iron (yes/no) and current eGFR between 15 and 45 mL/min/1.73 m^2^ or 46 and 59 mL/min/1.73 m^2^] as factors and baseline Hb as covariate.

^b^The first P-value represents the non-inferiority test and the second P-value represents the superiority test.

**FIGURE 3:**
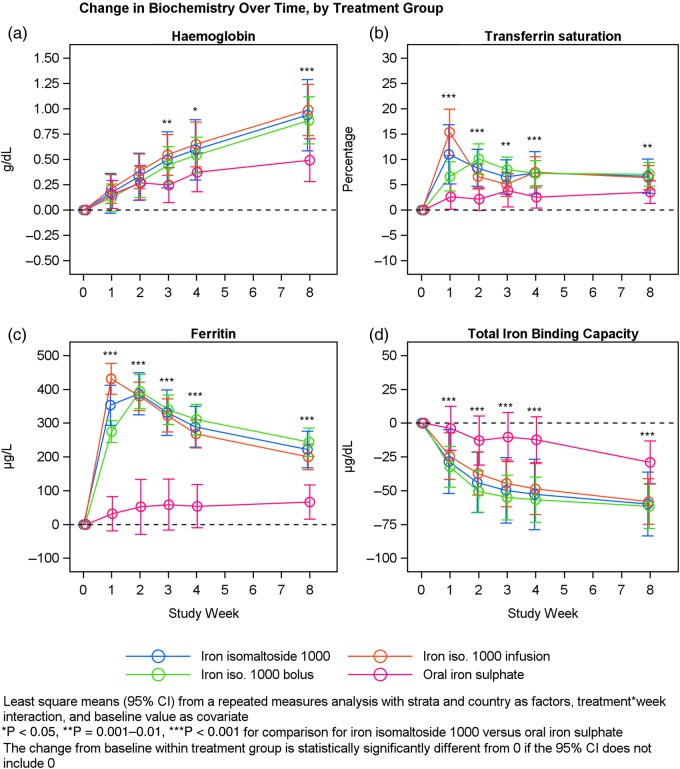
Hb, serum ferritin, TSAT and TIBC over time by treatment group.

There was a statistically significant larger increase in Hb concentration from baseline to Week 8 within Group A compared with Group B (P < 0.001) (Table 4 and Figure 3). Similar results were observed when Groups A1 and A2 were compared with Group B (Supplementary Tables S1 and S2).

**FIGURE 4:**
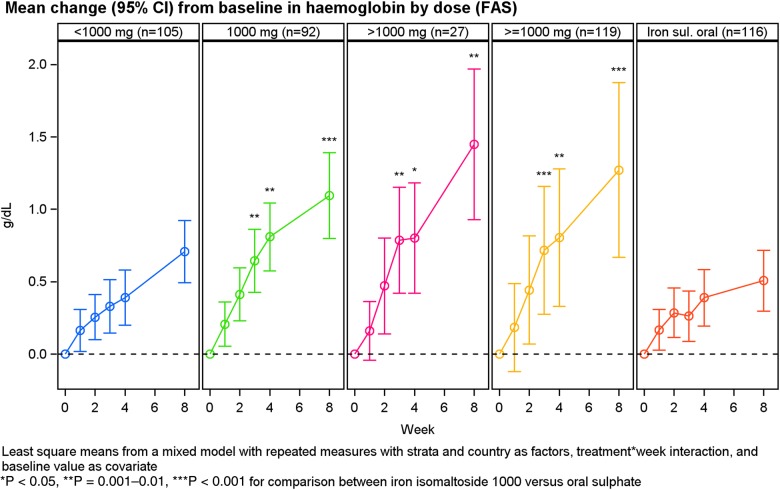
Hb over time by dose, mean (±SD) baseline Hb was 10.09 ± 1.03, 9.56 ± 0.92, 8.39 ± 1.13 and 9.64 ± 1.05 g/dL for patients treated with <1000 mg, 1000 mg, >1000 mg iron isomaltoside 1000 and oral iron sulphate, respectively.

An exploratory analysis showed that the significantly superior increase in Hb concentration with iron isomaltoside 1000 compared with oral iron sulphate was sustained from Week 3 until Week 8 with a gradual increase throughout Weeks 1–8 (Figure 3). In addition, the Hb response was more pronounced with iron isomaltoside 1000 doses of 1000 mg or greater (Figure 4).

#### Change in other laboratory parameters

Secondary outcomes included change in other laboratory parameters and this was conducted on the FAS (*N* = 340).

The estimated effect size for the laboratory parameters, including precision, is shown for Group A compared with B in Table 4, Group A1 compared with B in Supplementary Table S1 and Group A2 compared with B in Supplementary Table S2.

#### Change in concentrations of serum-iron, serum-ferritin, TSAT and TIBC

There was a statistically significant larger increase in serum iron concentration from baseline to Week 1 and Week 2 in Group A compared with Group B (Week 1: P < 0.001; Week 2: P = 0.003) (Table 4). Similar results were observed when Group A1 was compared with Group B (Supplementary Table S1) and when Group A2 was compared with Group B at Week 2 (Supplementary Table S2).

There was a statistically significant larger increase in serum-ferritin concentration and TSAT, and larger decrease in TIBC, from baseline to Weeks 1, 2, 4 and 8 in Group A compared with Group B (P < 0.001 for serum ferritin and TIBC at all time points and for TSAT at Weeks 1–4; P = 0.004 for TSAT at Week 8) (Table 4 and Figure 3). Similar results were observed for all iron parameters when Groups A1 and A2 were compared with Group B (Supplementary Tables S1 and S2).

#### Change in total QoL

The change in QoL (secondary outcomes) was assessed in the FAS (*N* = 340).

There was a statistically significant improvement in QoL (energy level, ability to do daily activities and overall QoL) from baseline to Week 8 within each treatment group. The improvement in QoL was similar in Groups A and B and there were no statistical difference between them (Supplementary Table S3).

### Safety

All safety analyses were conducted on the safety analysis set (*N* = 345).

There was no statistical significant difference in the proportion of patients experiencing an adverse event (AE) between Groups A and B [Group A: 95/228 (41.7%); Group B: 53/117 (45.3%)] (Table 5). There were a total of 86 treatment emergent AEs in 39/116 patients (33.6%) in Group A1 and 100 treatment emergent AEs in 56/112 patients (50.0%) in Group A2 (P = 0.02). The main reason for this difference was a higher frequency of pyrexia and infections such as gastroenteritis, rhinitis and respiratory tract infections in Group A2 patients.

**Table 5.** Summary of adverse events for iron isomaltoside 1000 (infusion and bolus dose) and iron sulphate, safety population

Number of patients	Iron isomaltoside 1000, infusion (*n* = 116)	Iron isomaltoside 1000, bolus (*n* = 112)	Iron sulphate (*n* = 117)
Adverse events, *n* (%)	39 (33.6)	56 (50.0)	53 (45.3)
ADR, *n* (%)	10 (8.6)	14 (12.5)	12 (10.3)
Serious adverse events, *n* (%)	6 (5.2)	6 (5.4)	10 (8.5)
Serious ADRs, *n* (%)	1 (0.9)	1 (0.9)	1 (0.9)
Suspected unexpected SAR, *n* (%)	–	–	–
Withdrawals due to adverse events	1 (0.9)	1 (0.9)	5 (4.3)

In relation to severity, causality and outcome, the AEs were comparable between the groups, and the majority of the AEs were mild or moderate and unrelated to the trial drug as adjudged by the trial safety committee after review of the temporal relationship of the AE with iron dosing as well as the comments of the local principal investigator.

ADRs (i.e. treatment-related AEs) were observed in 24/228 (10.5%) of the patients in Group A and in 12/117 (10.3%) of the patients in Group B. No dose relationship was found. A total of 14 ADR events were classified as related to trial therapy in 10/116 (8.6%) patients in Group A1 and there were 19 events in 14/112 (12.5%) patients in Group A2. There was no significant difference in the number of ADRs between Groups A1 and A2 (P = 0.39).

Three SARs [two events of hypersensitivity in Group A (2/228, 0.9%) and one event of oesophagitis in Group B (1/117, 0.9%)] were reported. The first event of hypersensitivity occurred during the first exposure to iron isomaltoside 1000 in a patient with a medical history of ischaemic heart disease. The patient had a marked decrease in blood pressure which required treatment with atropine and adrenaline. The other hypersensitivity event occurred in a patient during the second administration of iron isomaltoside 1000 after the first dosage had been well tolerated. No hypotension was reported in this patient and the patient recovered following treatment with steroid only. Both patients fully recovered from the events.

There were three fatal events during the study, all occurring in patients in Group A. None of the fatal events were related to iron isomaltoside 1000, and all three subjects had a significant prior history of cardiac disease. Two 82-year-old men died of decompensated heart failure 3 months and 6 weeks, respectively, after receiving the study drug [one had previous myocardial infarction (MI), diabetes, heart failure and peripheral vascular bypass; the other patient had previous heart failure and ventricular arrhythmias]. An 84-year-old woman with previous coronary bypass, pacemaker and diabetes developed pneumonia and died of MI 6 days after an iron bolus treatment.

More patients treated with oral iron sulphate were withdrawn from the study due to AEs (5/117, 4.3%) than patients treated with iron isomaltoside 1000 (2/228, 0.9%).

Transient hypophosphataemia (phosphate <2 mg/dL) was reported in five patients [Group A1: 3/116 (2.6%); Group A2: 1/112 (0.9%); Group B: 1/117 (0.9%)]. The three patients in Group A1 had a phosphate level of 1.2, 1.7 and 1.8 mg/dL, and both patients in Groups A2 and B had a phosphate level of 1.2 mg/dL. None of these hypophosphataemic events were reported as AEs.

## DISCUSSION

The objective of the trial was to evaluate the efficacy and short-term safety of IV iron isomaltoside 1000 administered by infusions or repeated bolus injections in comparison with oral iron sulphate in NDD-CKD patients with renal-related anaemia. Iron sulphate was selected as the comparator in this study since it has been widely used in the treatment of iron deficiency anaemia in previous studies in CKD patients [10, 11]. In the present trial, IV iron isomaltoside 1000 was not only non-inferior but also superior to iron sulphate in achieving the primary outcome. It induced a significantly higher increase in the level of Hb from baseline to Week 4 than twice daily administration of 100 mg iron sulphate, and this was sustained over an 8-week treatment period. This indicates a greater efficacy of iron isomaltoside 1000 over oral iron. The superior effect was sustained from Week 3 and until the end of the study at Week 8. Furthermore, the Hb response was more pronounced with iron isomaltoside 1000 doses ≥1000 mg. Similar results have been observed in other recent studies with CKD patients [10, 11]. In the study by Qunibi *et al.* [11], IV ferric carboxymaltose was found to induce a significantly higher increase in Hb when compared with oral iron over an 8-week treatment period in NDD-CKD patients, and in a study by Spinowitz *et al.* [10], IV ferumoxytol led to a significantly higher increase in Hb from baseline to Day 35 when compared with oral iron in CKD patients. Both of these studies utilized higher cumulative mean doses of IV iron than were administered in our trial and allowed patients to be on an ESA, whereas in our trial, NDD-CKD patients were ESA naive. At the current time, the optimal Hb target for NDD-CKD patients who are not receiving an ESA has yet to be established and this issue merits further investigation.

In the present trial, there was a statistically significant increase in serum ferritin and TSAT concentration and a decrease in TIBC concentration from baseline to Week 8 in patients treated with iron isomaltoside 1000. These results potentially signify a faster repletion of body iron stores with administration of iron isomaltoside 1000 than with iron sulphate, an effect also noted in the trial by Qunibi *et al.* [11] with IV ferric carboxymaltose.

Even though there was a statistically significant improvement in QoL within each treatment group, there was no difference between patients treated with either iron isomaltoside 1000 or oral iron sulphate. Thus, the clinical effect of the statistically higher increase in Hb and iron-related parameters related to the IV iron treatment needs to be evaluated further; it is unknown whether an increase in Hb of <1 g/dL will have any effect on QoL.

The majority of the AEs in both treatment groups were mild or moderate and unrelated to the trial drug. The frequency of ADRs was comparable between the treatment groups (10.5 versus 10.3%). Three SARs (two events of hypersensitivity in Group A and one event of oesophagitis in Group B) were reported. Both patients recovered from the hypersensitivity events. Although there were three fatal events, these were due to cardiac events and all occurred in patients with prior significant cardiac disease, there being no relationship with the study drug. The safety findings were comparable with other trials investigating iron isomaltoside 1000 that have shown a good safety profile in CKD patients [4, 12, 13].

More patients treated with oral iron sulphate were withdrawn from the study due to AEs than patients treated with iron isomaltoside 1000 (4.3 versus 0.9%). One limitation was that the modified MDRD formula for Asian ethnicity was not utilized within the study; it is recognized that the standard MDRD formula will over-estimate eGFR in Asians and this may have been the case in this study. However, the mean eGFR and the proportion of Asian and European patients were very similar between the treatment groups. Although no specific ethnicity subgroup analysis was performed to assess whether changes in Hb or iron parameters were different between Asians and Europeans, the country in which patients were recruited was included in the multi-variable analysis, and hence, we feel that the study results are generalizable to a wide multiracial population.

In conclusion, this randomized trial demonstrated that iron isomaltoside 1000 is superior to oral iron sulphate in its ability to increase Hb over an 8-week period. Markers of iron deficiency were also significantly improved. The safety profile of iron isomaltoside 1000 was comparable with oral iron sulphate in patients with NDD-CKD and renal-related anaemia, but more patients stopped oral therapy due to side effects.

## SUPPLEMENTARY DATA

Supplementary data are available online at http://ndt.oxfordjournals.org.

## AUTHORS’ CONTRIBUTIONS

Research idea and trial design: P.A.K., S.B., L.L.T. and D.W.C.; data acquisition, data analysis and interpretation: P.A.K., S.B., S.S, D.A., G.W., J.K., L.L.T. and D.W.C. Each author was involved in revising the manuscript critically for important intellectual content, and all authors read and approved the final manuscript.

## CONFLICT OF INTEREST STATEMENT

All investigators/institutions received fees for patient enrollment. P.A.K. has received speaker and consultancy fees and assistance with travel from Pharmacosmos A/S, Vifor and Takeda. S.B. has received speaker and consultancy fees from Pharmacosmos A/S. S. Saxena, D. Agarwal, G. Wirtz and J. Kletzmayr have no further conflict of interest. L.L.T. is employed by Pharmacosmos A/S. D.W.C. is a consultant to Pharmacosmos A/S, Vifor and Keryx and was previously a consultant and speaker for Watson (now Actavis) and Sanofi Aventis. The trial was funded by Pharmacosmos A/S.

## Supplementary Material

Supplementary Data

## References

[1] KDIGO Clinical Practice Guideline for Anemia in Chronic Kidney Disease. *Kidney Int Suppl* 2012; 2: 279–335

[2] Charytan C, Qunibi W, Bailie GR Comparison of intravenous iron sucrose to oral iron in the treatment of anemic patients with chronic kidney disease not on dialysis. *Nephron Clin Pract* 2005; 100: c55–c62 15824508 10.1159/000085049

[3] Macdougall IC, Bock AH, Carrera F et al FIND-CKD: a randomized trial of intravenous ferric carboxymaltose versus oral iron in patients with chronic kidney disease and iron deficiency anaemia. *Nephrol Dial Transplant* 2014; 29: 2075–2084 24891437 10.1093/ndt/gfu201 PMC4209879

[4] Wikstrom B, Bhandari S, Barany P et al Iron isomaltoside 1000: a new intravenous iron for treating iron deficiency in chronic kidney disease. *J Nephrol* 2011; 24: 589–596 21240875 10.5301/JN.2011.6248

[5] Hildebrandt PR, Bruun NE, Nielsen OW et al Effects of administration of iron isomaltoside 1000 in patients with chronic heart failure. A pilot study. *TATM* 2010; 11: 131–137

[6] Reinisch W, Staun M, Tandon RK et al A randomized, open-label, non-inferiority study of intravenous iron isomaltoside 1,000 (Monofer) compared with oral iron for treatment of anemia in IBD (PROCEED). *Am J Gastroenterol* 2013; 108: 1877–1888 24145678 10.1038/ajg.2013.335 PMC3853365

[7] Ganzoni AM Intravenous iron-dextran: therapeutic and experimental possibilities. *Schweiz Med Wochenschr* 1970; 100: 301–303 5413918

[8] Coates A, Dillenbeck CF, McNeil DR et al On the receiving end—II. Linear analogue self-assessment (LASA) in evaluation of aspects of the quality of life of cancer patients receiving therapy. *Eur J Cancer Clin Oncol* 1983; 19: 1633–1637 6315445 10.1016/0277-5379(83)90096-2

[9] Levey AS, Coresh J, Greene T et al Expressing the modification of diet in renal disease study equation for estimating glomerular filtration rate with standardized serum creatinine values. *Clin Chem* 2007; 53: 766–772 17332152 10.1373/clinchem.2006.077180

[10] Spinowitz BS, Kausz AT, Baptista J et al Ferumoxytol for treating iron deficiency anemia in CKD. *J Am Soc Nephrol* 2008; 19: 1599–1605 18525001 10.1681/ASN.2007101156 PMC2488268

[11] Qunibi WY, Martinez C, Smith M et al A randomized controlled trial comparing intravenous ferric carboxymaltose with oral iron for treatment of iron deficiency anaemia of non-dialysis-dependent chronic kidney disease patients. *Nephrol Dial Transplant* 2011; 26: 1599–1607 20929915 10.1093/ndt/gfq613 PMC3084440

[12] Aronoff GR, Bennett WM, Blumenthal S et al Iron sucrose in hemodialysis patients: safety of replacement and maintenance regimens. *Kidney Int* 2004; 66: 1193–1198 15327417 10.1111/j.1523-1755.2004.00872.x

[13] Gupta DR, Larson DS, Thomsen LL et al Pharmacokinetics of iron iromaltoside 1000 in patients with stage 5 chronic kidney disease on dialysis therapy. *J Drug Metab Toxicol* 2013; 4 doi:10.4172/2157-7609.1000152

