# Supplementary material for: A randomized trial of iron isomaltoside 1000 versus oral iron in non-dialysis-dependent chronic kidney disease patients with anaemia
Source: Nephrol Dial Transplant. 2015 Aug 6;31(4):646–55. doi: 10.1093/ndt/gfv293 (PMC4805129; doi:10.1093/ndt/gfv293)
Supplement: Supplementary Data [file supp_31_4_646__index.html]

A randomized trial of iron isomaltoside 1000 versus oral iron in non-dialysis-dependent chronic kidney disease patients with anaemia — A randomized trial of iron isomaltoside 1000 versus oral iron in non-dialysis-dependent chronic kidney disease patients with anaemia — Supplementary Data 

# A randomized trial of iron isomaltoside 1000 versus oral iron in non-dialysis-dependent chronic kidney disease patients with anaemia

## Supplementary Data

Supplementary Data

- Supplementary Data - Docx file
