# Supplementary material for: A randomized trial of iron isomaltoside 1000 versus oral iron in non-dialysis-dependent chronic kidney disease patients with anaemia
Source: Nephrol Dial Transplant. 2015 Aug 6;31(4):646–55. doi: 10.1093/ndt/gfv293 (PMC4805129; doi:10.1093/ndt/gfv293)
Supplement: Supplementary Data [file supp_gfv293_gfv293supp.docx]

**Supplementary tables**

**Table S1** Laboratory parameters: estimated effect size and its precision, group A1 versus B

| **Laboratory parameter, time point (number of patients)** | **Iron isomaltoside 1000 (group A1), least-square mean estimate*** | **Iron sulphate (group B), least-square mean estimate*** | **Difference estimates (95% CI)** | ***P* value** |
| --- | --- | --- | --- | --- |
| **Haemoglobin (g/dL) – full analysis set** | | | | |
| Week 2 (group A1: 105, group B: 110) | 0.38 | 0.27 | 0.11 (-0.085;0.31 ) | 0.26 |
| Week 4 (group A1: 109, group B: 108) | 0.65 | 0.37 | 0.27 (0.015: 0.53) | <0.001/0.038** |
| Week 8 (group A1: 106, group B: 112) | 0.99 | 0.49 | 0.50 (0.20;0.80) | 0.001 |
| **Haemoglobin (g/dL) – per protocol analysis set** | | | | |
| Week 4 (group A1: 107, group B: 106) | 0.66 | 0.39 | 0.27 (0.007;0.54) | <0.001/0.044** |
| **Serum-iron (µg/dL) – full analysis set** | | | | |
| Week 1 (group A1: 109, group B: 109) | 8.70 | 2.66 | 6.04 (3.92;8.15) | <0.001 |
| Week 2 (group A1: 104, group B: 110) | 3.76 | 2.02 | 1.74 (0.28;3.20) | 0.020 |
| Week 4 (group A1: 108, group B: 108) | 3.04 | 1.99 | 1.05 (-0.33;2.43) | 0.14 |
| Week 8 (group A1: 106, group B: 112) | 3.00 | 1.86 | 1.14 (-0.13;2.40) | 0.079 |
| **Serum-ferritin (ng/mL) – full analysis set** | | | | |
| Week 1 (group A1: 109, group B: 109) | 431 | 32 | 400 (340;460) | <0.001 |
| Week 2 (group A1: 104, group B: 110) | 380 | 52 | 328 (243;413) | <0.001 |
| Week 4 (group A1: 108, group B: 108) | 268 | 54 | 214 (145;283) | <0.001 |
| Week 8 (group A1: 106, group B: 112) | 200 | 66 | 134 (80;188) | <0.001 |
| **Transferrin saturation (%) – full analysis set** | | | | |
| Week 1 (group A1: 109, group B: 109) | 15.36 | 2.64 | 12.72 (7.98;17.47) | <0.001 |
| Week 2 (group A1: 104, group B: 110) | 6.64 | 2.14 | 4.50 (1.96;7.03) | <0.001 |
| Week 4 (group A1: 108, group B: 108) | 7.44 | 2.57 | 4.87 (1.80;7.94) | 0.002 |
| Week 8 (group A1: 106, group B: 112) | 6.39 | 3.54 | 2.86 (0.38;5.34) | 0.024 |
| **Total iron binding capacity (µmol/L) – full analysis set** | | | | |
| Week 1 (group A1: 109, group B: 109) | -4.67 | -0.72 | -3.95 (-5.97;-1.93) | <0.001 |
| Week 2 (group A1: 104, group B: 110) | -7.24 | -2.33 | -4.91 (-6.91;-2.91) | <0.001 |
| Week 4 (group A1: 108, group B: 108) | -10.13 | -2.70 | -7.43 (-9.90;-4.96) | <0.001 |
| Week 8 (group A1: 106, group B: 112) | -9.87 | -5.19 | -4.68 (-6.52;-2.84) | <0.001 |

* Least-square means from repeated measures model with treatment, visit, treatment*visit interactions, country, and stratum (past treatment with parenteral iron (Yes/No) and current eGFR between 15-45 mL/min or between 46-59 mL/min) as factors and baseline Hb as covariate.

**The first p-value represents the non-inferiority test and the second p-value represents the superiority test.

Conversion factor for serum-iron: µmol/L / 0.179 = µg/dL

**Table S2** Laboratory parameters: estimated effect size and its precision, group A2 versus B.

| **Laboratory parameter, time point (number of patients)** | **Iron isomaltoside 1000 (group A2), least-square mean estimate*** | **Iron sulphate (group B), least-square mean estimate*** | **Difference estimates (95% CI)** | ***P* value** |
| --- | --- | --- | --- | --- |
| **Haemoglobin (g/dL) – full analysis set** | | | | |
| Week 2 (group A2: 105, group B: 110) | 0.28 | 0.27 | 0.0065 (-0.18;0.19) | 0.94 |
| Week 4 (group A2: 100, group B: 108) | 0.54 | 0.37 | 0.17 (-0.055: 0.39) | <0.001/0.14** |
| Week 8 (group A2: 104, group B: 112) | 0.89 | 0.49 | 0.39 (0.11;0.68) | 0.007 |
| **Haemoglobin (g/dL) – per protocol analysis set** | | | | |
| Week 4 (group A2: 97, group B: 106) | 0.55 | 0.39 | 0.16 (-0.066;0.39) | <0.001/0.16** |
| **Serum-iron (µg/dL) – full analysis set** | | | | |
| Week 1 (group A2: 108, group B: 109) | 3.73 | 2.66 | 1.07 (-0.86;3.01) | 0.28 |
| Week 2 (group A2: 105, group B: 110) | 4.35 | 2.02 | 2.32 (0.78;3.86) | 0.003 |
| Week 4 (group A2: 100, group B: 108) | 2.74 | 1.99 | 0.75 (-0.61;2.10) | 0.28 |
| Week 8 (group A2: 103, group B: 112) | 2.63 | 1.86 | 0.77 (-0.49;2.02) | 0.23 |
| **Serum-ferritin (ng/mL) – full analysis set** | | | | |
| Week 1 (group A2: 108, group B: 109) | 275 | 32 | 243 (192;294) | <0.001 |
| Week 2 (group A2: 105, group B: 110) | 394 | 52 | 342 (252;432) | <0.001 |
| Week 4 (group A2: 100, group B: 108) | 310 | 54 | 256 (185;328) | <0.001 |
| Week 8 (group A2: 103, group B: 112) | 244 | 66 | 177 (120;234) | <0.001 |
| **Transferrin saturation (%) – full analysis set** | | | | |
| Week 1 (group A2: 108, group B: 109) | 6.65 | 2.64 | 4.02 (0.86;7.17) | 0.013 |
| Week 2 (group A2: 105, group B: 110) | 10.05 | 2.14 | 7.91 (4.83;10.99) | <0.001 |
| Week 4 (group A2: 100, group B: 108) | 7.24 | 2.57 | 4.67 (2.12;7.21) | <0.001 |
| Week 8 (group A2: 103, group B: 112) | 7.08 | 3.54 | 3.54 (1.10;5.98) | 0.005 |
| **Total iron binding capacity (µmol/L) – full analysis set** | | | | |
| Week 1 (group A2: 108, group B: 109) | -4.61 | -0.72 | -3.89 (-5.40;-2.39) | <0.001 |
| Week 2 (group A2: 105, group B: 110) | -7.79 | -2.33 | -5.46 (-7.41;-3.51) | <0.001 |
| Week 4 (group A2: 100, group B: 108) | -9.58 | -2.70 | -6.88 (-8.82;-4.95) | <0.001 |
| Week 8 (group A2: 103, group B: 112) | -10.07 | -5.19 | -4.88 (-6.73;-3.02) | <0.001 |

* Least-square means from repeated measures model with treatment, visit, treatment*visit interactions, country, and stratum (past treatment with parenteral iron (Yes/No) and current eGFR between 15-45 mL/min or between 46-59 mL/min) as factors and baseline Hb as covariate.

**The first p-value represents the non-inferiority test and the second p-value represents the superiority test.

Conversion factor for serum-iron: µmol/L / 0.179 = µg/dL

**Table S3** Quality of life: estimated effect size and its precision, group A versus B, full analysis set

| **Quality of life (number of patients)** | **Iron isomaltoside 1000 (group A), least-square mean estimate* (standard error)** | **Iron sulphate (group B), least-square mean estimate* (standard error)** | **Difference estimates (95% CI)** | ***P* value** |
| --- | --- | --- | --- | --- |
| **Energy level** | | | | |
| Week 4 (group A: 194, group B: 103) | 8.4 (3.17) | 7.59 (2.1) | 0.81 (-3.00;4.62) | 0.68 |
| *P* value within group | 0.009 | <0.001 |  |  |
| Week 8 (group A: 204, group B: 108) | 12.2 (3.16) | 12.1 (2.19) | 0.096 (-3.93;4.12) | 0.96 |
| *P* value within group | <0.001 | <0.001 |  |  |
| **Ability to do daily activities** | | | | |
| Week 4 (group A: 194, group B: 103) | 7.08 (3.19) | 5.03 (1.98) | 2.04 (-1.67;5.75) | 0.28 |
| *P* value within group | 0.028 | 0.012 |  |  |
| Week 8 (group A: 204, group B: 108) | 10.0 (3.01) | 8.03 (2.2) | 2.02 (-2.06;6.09) | 0.33 |
| *P* value within group | 0.001 | <0.001 |  |  |
| **Overall quality of life** | | | | |
| Week 4 (group A: 194, group B: 103) | 4.38 (3.04) | 2.8 (1.94) | 1.58 (-2.04;5.20) | 0.39 |
| *P* value within group | 0.15 | 0.15 |  |  |
| Week 8 (group A: 204, group B: 108) | 7.56 (3.1) | 6.11 (2.12) | 1.46 (-2.53;5.45) | 0.47 |
| *P* value within group | 0.016 | 0.005 |  |  |

* Least-square means from repeated measures model with treatment, visit, treatment*visit interactions, country, and stratum (past treatment with parenteral iron (Yes/No) and current eGFR between 15-45 mL/min or between 46-59 mL/min) as factors and baseline Hb as covariate.
